# Fluorescent Graphitic Carbon Nitride (g-C_3_N_4_)-Embedded Hyaluronic Acid Microgel Composites for Bioimaging and Cancer-Cell Targetability as Viable Theragnostic

**DOI:** 10.3390/ph17020160

**Published:** 2024-01-25

**Authors:** Selin S. Suner, Mehtap Sahiner, Sahin Demirci, Evrim Umut, Nurettin Sahiner

**Affiliations:** 1 Department of Chemistry, Faulty of Science, Canakkale Onsekiz Mart University, 17100 Canakkale, Turkey; sagbasselin@gmail.com (S.S.S.); sahindemirci@gmail.com (S.D.); 2 Department of Bioengineering, Faculty of Engineering, Canakkale Onsekiz Mart University Terzioglu Campus, 17100 Canakkale, Turkey; sahinerm78@gmail.com; 3 Department of Medical Imaging Techniques, School of Healthcare, Dokuz Eylul University, 35330 Izmir, Turkey; evrim.umut@deu.edu.tr; 4 BioIzmir-Izmir Health Technologies Development and Accelerator Research and Application Center, Dokuz Eylul University, 35330 Izmir, Turkey; 5 Department of Chemical and Biomolecular Engineering, University of South Florida, Tampa, FL 33620, USA; 6 Department of Ophthalmology, Morsani College of Medicine, University of South Florida, Tampa, FL 33612, USA

**Keywords:** hyaluronic acid (HA), graphitic carbon nitride (g-C_3_N_4_), fluorescent–HA, fluorescence bioimaging, cancer-cell target

## Abstract

Fluorescent graphitic carbon nitride (g-C_3_N_4_) doped with various heteroatoms, such as B, P, and S, named ^B^g-C_3_N_4_, ^P^g-C_3_N_4_, and ^S^g-C_3_N_4_, were synthesized with variable band-gap values as diagnostic materials. Furthermore, they were embedded within hyaluronic acid (HA) microgels as g-C_3_N_4_@HA microgel composites. The g-C_3_N_4_@HA microgels had a 0.5–20 μm size range that is suitable for intravenous administration. Bare g-C_3_N_4_ showed excellent fluorescence ability with 360 nm excitation wavelength and 410–460 emission wavelengths for possible cell imaging application of g-C_3_N_4_@HA microgel composites as diagnostic agents. The g-C_3_N_4_@HA-based microgels were non-hemolytic, and no clotting effects on blood cells or cell toxicity on fibroblasts were observed at 1000 μg/mL concentration. In addition, approximately 70% cell viability for SKMEL-30 melanoma cells was seen with ^S^g-C_3_N_4_ and its HA microgel composites. The prepared g-C_3_N_4_@HA and ^S^g-C_3_N_4_@HA microgels were used in cell imaging because of their excellent penetration capability for healthy fibroblasts. Furthermore, g-C_3_N_4_-based materials did not interact with malignant cells, but their HA microgel composites had significant penetration capability linked to the binding function of HA with the cancerous cells. Flow cytometry analysis revealed that g-C_3_N_4_ and g-C_3_N_4_@HA microgel composites did not interfere with the viability of healthy fibroblast cells and provided fluorescence imaging without any staining while significantly decreasing the viability of cancerous cells. Overall, heteroatom-doped g-C_3_N_4_@HA microgel composites, especially ^S^g-C_3_N_4_@HA microgels, can be safely used as multifunctional theragnostic agents for both diagnostic as well as target and treatment purposes in cancer therapy because of their fluorescent nature.

## 1. Introduction

In the last decade, graphitic carbon nitride (g-C_3_N_4_), possessing a graphene-like two-dimensional sheet structure composed of heptazine (tri-s-triazine) units, has attracted tremendous attention owing to its photocatalytic properties. As a metal-free semiconductor, under UV–visible light that is larger than its band gap (hυ > Eg), g-C_3_N_4_ exhibits photoconductivity and fluorescence radiation and generates radicals. This can lead to new avenues for many energy applications, including hydrogen production, organic photovoltaics, ion transport membranes, and environmental applications such as photoinduced CO_2_ reduction and water pollutant degradation [1,2]. Although bulk g-C_3_N_4_ can be synthesized from nitrogen-rich precursors under mild conditions, disadvantages in terms of its physicochemical properties include poor dispersibility, low specific surface area, abundant surface defects, and fast recombination of photoexcited electron–hole pairs. All of these drawbacks hamper its photocatalytic performance [3]. In most applications, polymer composites incorporated with g-C_3_N_4_ are used, in which these materials are synergistically combined to fortify their mutual properties. For example, the construction of three-dimensional porous polymeric networks, i.e., hydrogels, either by photopolymerization using g-C_3_N_4_ as the photo-initiator in the presence of monomers or by embedding g-C_3_N_4_ into existing crosslinked polymers, enhances the mechanical stability (e.g., stretchability/processibility) of the hydrogels in addition to providing them with photocatalytic activity. Similarly, the gel networks serve as perfect host materials in which the g-C_3_N_4_ sheets can be dispersed homogeneously, rendering a higher specific surface area; this promotes photoactivity with an increased number of light absorption/emission sites [4,5,6].

Another way to improve the photocatalytic activity/photosensitivity of g-C_3_N_4_ in order to facilitate most of the abovementioned applications is the introduction of foreign nonmetal elements such as oxygen (O), halogens (X:Cl, F, etc.), phosphorus (P), sulfur (S), and boron (B) into the g-C_3_N_4_ structure [7,8,9]. These heteroatoms may replace an existing atom in the lattice (substitutional doping) or can be in the planar cavity (interstitial doping) of g-C_3_N_4_. This can be achieved by hybridization of molecular orbitals with a doped orbital, changing the electronic properties of g-C_3_N_4_. As a result, the valence and conduction band energies shift towards each other, narrowing the band gap of doped g-C_3_N_4_ (typically Eg = 2.7 eV) and enhancing light absorption. Doping atoms can also create impurity states (levels) in the band gap at which the photogenerated charge carriers (electrons and holes) localize and jump back to the corresponding bands rather than undergo recombination; this increases the photoinduced redox performance of doped g-C_3_N_4_ [10]. 

The pioneering study in 2009 by Wang et al. showed that hydrogen generation by water splitting under visible light is possible with polymeric carbon nitride [11]. Following this, there has been growing interest in using g-C_3_N_4_/polymer composites in fuel cells, CO_2_ reduction, and organic pollutant degradation. However, their use in the biomedical field, e.g., in terms of imaging and treatment capabilities by means of g-C_3_N_4_, has not been fully explored. In photodynamic therapy (PDT), for example, g-C_3_N_4_ may be beneficial as a photosynthesizer, which releases reactive oxygen species (ROS) that can induce a path for the destruction of tumor cells after UV–visible light exposure [12]. This therapeutic effect can be combined with imaging capabilities facilitated by photoluminescence emitted by g-C_3_N_4_/polymer composites. These types of versatile material diagnostic and phototherapy methods, including biomedical imaging, are very much needed. Liu et al. prepared drug-loaded hyaluronic acid (HA)-modified hollow g-C_3_N_4_ nanospheres and demonstrated that effective cancer treatment is possible via light-excitation-triggered drug release following the cellular uptake of these nanospheres [13]. In another study, the feasibility of simultaneous two-photon imaging and PDT application was reported using single-component g-C_3_N_4_ quantum dots obtained by introducing active sites of a disordered structure of defects to pristine g-C_3_N_4_ [14].

In this paper, we present the preparation of heteroatom (B, P, and S)-doped g-C_3_N_4,_ as ^B^g-C_3_N_4_-, ^P^g-C_3_N_4_-, and ^S^g-C_3_N_4_-embedded hyaluronic acid (HA) microgels. These can serve as fluorescent probes in bioimaging applications and have specific targeting ability via the binding capability of HA for some specific receptors on tumor cells. For this purpose, HA microgels are a perfectly biocompatible material as they are innately present in skin, tears, joints, and soft connective tissues, and a component of the extracellular matrix [15], which can be exploited by preparing them at sizes d < 20 μm. HA is known to have a specific binding ability against CD44 [16,17] receptor HA-mediated motility (RHAMM), P-selectin [18], and lymphatic vessel endocytic receptor (LYVE-1) receptors, which are commonly present in various cancer cells [19]. Therefore, HA microgels embedding g-C_3_N_4_-based materials, which are fluorophores and photoactive, could be very useful for bioimaging applications and light-triggered disease treatments. As heteroatom-doped g-C_3_N_4_@HA-based microgel composites have strong fluorescence with UV–visible light exposure, they can be used for multiple purposes, e.g., imaging, targeting, and PDT. Although the tissue penetration depth of light is limited for UV–visible light, it may be proposed that under UV–visible light exposure, the synthesized heteroatom-doped g-C_3_N_4_@HA microgel can also be used for PDT for hypodermal tumors following transdermal introduction into the body. Therefore, the toxicities of g-C_3_N_4_ structures and g-C_3_N_4_@HA microgel composite were investigated by employing blood compatibility tests of hemolysis and blood clotting assays, as well as identifying cell toxicities for L929 fibroblast cells and SKMEL 30 human melanoma cells. The interactions of heteroatom-doped g-C_3_N_4_ and g-C_3_N_4_@HA microgel composites with L929 fibroblast cells and SK-MEL30 skin-cancer cells were investigated in detail to show the targeting ability of the prepared HA-based composites. 

## 2. Results and Discussion

**Figure 1:**
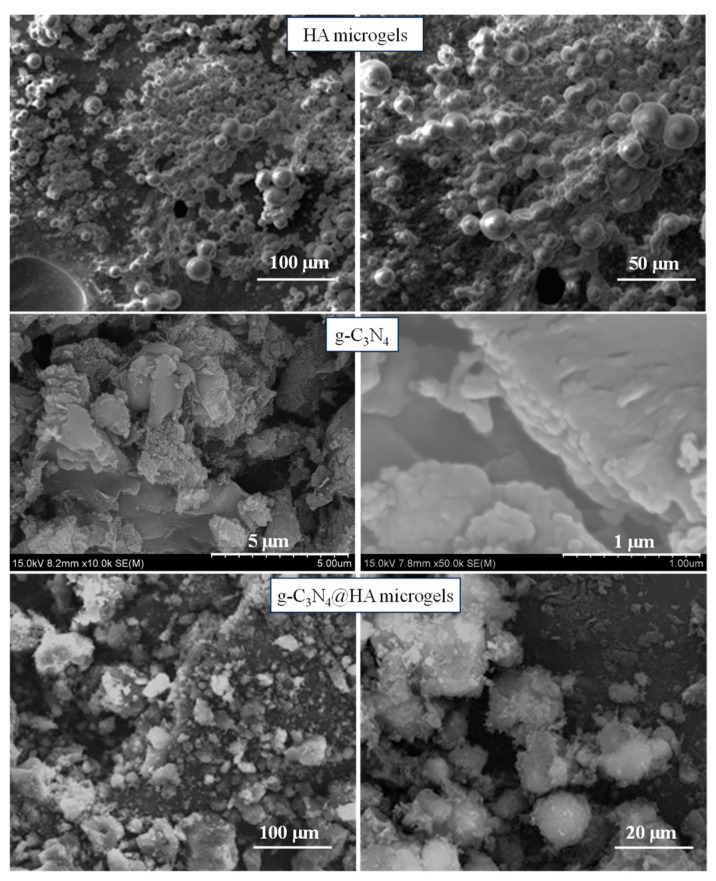
SEM images of HA microgels, g-C_3_N_4_, and g-C_3_N_4_@HA microgel composites.

The synthesis and characterization of g-C_3_N_4_ and heteroatom-doped ^H^g-C_3_N_4_ structures were reported previously [20]. The schematic presentation of the preparation of bare and heteroatom-doped ^H^g-C_3_N_4_ structures is also illustrated in Figure S1a. As can be clearly seen from Figure S1a, g-C_3_N_4_ structures were prepared from melamine by simple thermolysis and condensation reactions at 550 °C. The g-C_3_N_4_ generally consists of triazine (C_3_N_3_) or heptazine (C_6_N_7_) ring units [21,22]. Here, g-C_3_N_4_ structures were also doped with heteroatoms such as B, P, and S (^H^g-C_3_N_4_, H: B, P or S), using boric acid, phosphorus red, and sulfur as doping agents, respectively. The heteroatoms are anticipated to be randomly distributed throughout the network structure. In this study, the prepared g-C_3_N_4_ and ^H^g-C_3_N_4_ structures were integrated into HA microgels during the synthesis of the microgels, and the corresponding schematic presentation is illustrated in Figure S1b. In brief, 1 mL of HA solution in 0.1 M NaOH at 15 mg/mL concentration was added into 30 mL 0.2 M of AOT/isooctane. Also, at the same time 0.1 mL of g-C_3_N_4_-based suspension (15 mg/mL) was added to this solution and stirred for 15 min at room temperature to obtain a homogeneous mixture. After that, the crosslinker DVS was added to the mixture and stirred for 2 h at 1000 rpm to crosslink HA chains and embed g-C_3_N_4_-based structures into HA microgels. The digital camera images of the prepared HA and g-C_3_N_4_-embedded HA (g-C_3_N_4_@HA) microgels are also shown in Figure S1c. The change in the color of HA microgels from white to different tones of yellow after embedding g-C_3_N_4_ and Hg-C_3_N_4_ is clearly visible. Moreover, SEM images of HA microgels and g-C_3_N_4_@HA microgel composites are shown to present the morphological differences between bare and composite microgel forms. As seen in Figure 1, the spherical-shaped HA microgels with a size range of about 20 µm converted into irregular particles of g-C_3_N_4_@HA microgel composite due to the aggregates of g-C_3_N_4_ on and around the HA microgels. The micrometer size range of HA microgels ensures injectability by standard needles, which have 0.34–0.29 mm size range [23,24], or by an intravenous catheter with inner dimensions in the range from 1.6 mm to 0.5 mm [25].

As can be seen from the SEM images of g-C_3_N_4_, the nanosheets of g-C_3_N_4_ formed multiple thin layers stacked with large irregular block structures [26,27]. Similarly, the irregular stacks of aggregated g-C_3_N_4_ on HA particles were observed for the g-C_3_N_4_@HA microgel composite. Therefore, the aggregated g-C_3_N_4_ nanosheets inside, on, and around the HA microgels are due to the inherent block structure of g-C_3_N_4_ nanosheets. Furthermore, these images confirm that g-C_3_N_4_ was successfully embedded in the HA microgel network. 

The chemical composition of g-C_3_N_4_@HA, as an example of all prepared microgel composites, was verified by X-ray photoelectron spectroscopy (XPS). In the low-resolution survey spectra given in Figure S2a, as well as the labeled peaks for C1S, N1S, and O1S, more peaks belonging to Na1S and S2P atoms that are involved in the synthesis steps were identified. Since HA also includes carbon, nitrogen, and oxygen atoms, the relative intensities of the corresponding XPS peaks do not provide information about the atomic ratios of C:N:O in g-C_3_N_4_ alone. The high-resolution spectra for C1S peaks given in Figure S2b can be deconvoluted into three peaks, where the most intense peak observed at binding energy 284.5 eV originates from the graphitic phase in the aromatic ring. The other two components located at 286.1 eV–288 eV may be associated with C atoms in N=C-N and N-C-N groups, respectively. Consistently, in Figure S2c, the single peak observed for N1S at 399.4 eV can be assigned to sp^2^ hybridized N atoms in triazine rings.

Moreover, the FT-IR spectra for bare and g-C_3_N_4_-embedded HA microgels are given in Figure S3a to compare and confirm that g-C_3_N_4_ was embedded into HA microgels. Also, the thermograms for the bare and g-C_3_N_4_-embedded HA microgels are shown in Figure S3b. From the FT-IR spectra, the characteristic peaks for HA chains [28] were also observed in FT-IR spectra of bare and g-C_3_N_4_-embedded HA microgel composites as C=O for carboxylic acid at 1725 cm^−1^, C=O for the amide at 1605 cm^−1^, N-H stretching for amide groups at 1400 cm^−1^, and the C-O peaks for ether linkages at 1045 cm^−1^. The peaks at 1310 and 1170 cm^−1^ were assigned to symmetric and asymmetric S=O peaks coming from the crosslinker, DVS, respectively. However, no significant differences were observed between the FT-IR spectrum of bare and g-C_3_N_4_-embedded HA microgels. This could be due to the overlapping of peaks of the g-C_3_N_4_ structure [20] and HA microgels, in addition to the lower amount of g-C_3_N_4_ content in HA microgel composites (~10% max). In Figure S3b, the thermograms of HA-based microgels are compared to display the thermal stabilities of HA microgels with microgel composites containing different g-C_3_N_4_. It is clearly seen that all samples, bare and g-C_3_N_4_-embedded forms of HA microgels, exhibited similar thermal degradation profiles. The HA microgel, ^B^g-C_3_N_4_@HA, and ^S^g-C_3_N_4_@HA microgel structures were almost stable with no thermal degradation up to 260 °C, whereas almost 7% weight loss was observed at 260 °C for ^P^g-C_3_N_4_@HA microgels. Similar degradation steps in the 300–750 °C range with different weight loss% values were observed for all samples. Overall, the weight loss% values for HA microgel, ^B^g-C_3_N_4_@HA, ^P^g-C_3_N_4_@HA, and ^S^g-C_3_N_4_@HA microgels were determined as 65.1, 70.5, 64.5, 77.4, and 61.6%, respectively.

**Figure 2:**
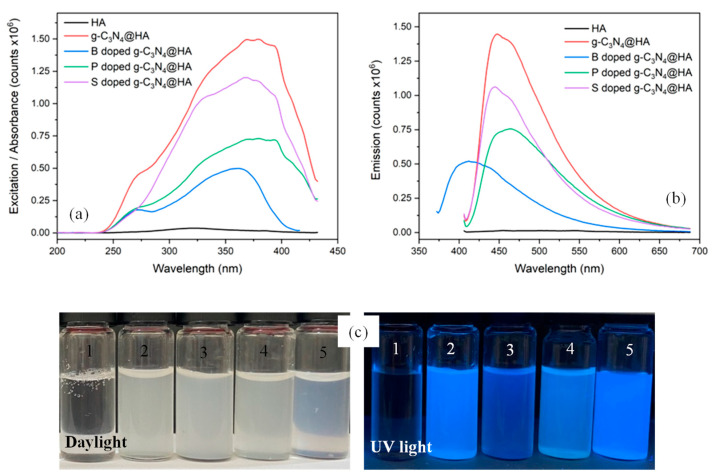
(**a**) Steady-state excitation/absorbance, (**b**) fluorescence emission spectra of aqueous suspensions of undoped and hetero atom doped ^H^g-C_3_N_4_@HA (H: B, S, P) microgel composites, and (**c**) digital camera images of HA and ^H^g-C_3_N_4_@HA microgel composites under daylight and UV light (λ = 365 nm) (1) HA microgels, (2) g-C_3_N_4_@HA microgels, (3) ^B^g-C_3_N_4_@HA microgels, (4) ^P^g-C_3_N_4_@HA microgels, and (5) ^S^g-C_3_N_4_@HA microgel composites.

The optical properties of aqueous dispersions of undoped and doped g-C_3_N_4_@HA microgels were investigated by fluorescence spectroscopy. In Figure 2a, HA-based microgels embedded with g-C_3_N_4_ exhibit wide absorption spectra in the UV range, reaching a maximum value at a wavelength of λ = 394 nm for undoped, S- and P-doped g-C_3_N_4_@HA microgels and λ = 360 nm for B-doped g-C_3_N_4_@HA microgel. Accordingly, when exposed to UV light at these wavelengths, microgels show fluorescent emissions in the visible range, as seen in Figure 2b. The g-C_3_N_4_ optical property was retained in the prepared microgel composites, even with slight enhancement. Since photoluminescence is a result of radiative recombination of charge carriers (electrons and holes) separated by the band gap Eg, using the formula Eg = h.c/λmax, where h is the Planck constant, c is the speed of light and λmax is the wavelength value for the emission spectra (Figure 2b), the band-gap energies can be roughly estimated as Eg = 2.75 eV for pristine g-C_3_N_4_, as Eg = 2.75 eV for ^S^g-C_3_N_4_, as Eg = 2.66 eV for ^P^g-C_3_N_4_, and 3.0 eV for ^B^g-C_3_N_4_@HA samples. This means S doping did not cause any significant change in the band gap of pristine g-C_3_N_4_, whereas the P-doped sample has a narrower band gap, as expected. Interestingly, B doping increased the band gap associated with the blue shift of the PL peak; similar observations were reported in other studies [29].

This phenomenon can be explained by the quantum confinement effect (which co-exists with band-gap narrowing), possibly originating from the decreased thickness of two-dimensional g-C_3_N_4_ nanosheets in this sample. Another observation in Figure 2b is that both absorption and emission peaks are weakened for doped samples, which is an indication that carrier recombination is suppressed (carrier lifetime is prolonged) since the doping with heteroatoms also serves as traps rather than as recombination centers for carriers. This is also favorable as the trapped carriers do not undergo radiative recombination and are expected to participate in reduction and oxidation reactions at the surface, promoting ROS generation. The optical activity of undoped and doped g-C_3_N_4_@HA microgel composites was further visualized with digital camera images under daylight and UV light (365 nm), as shown in Figure 2c. Comparing the digital camera images under daylight, no emission is observed. On the other hand, under UV light at 365 nm, these regions are easily distinguished, and the strong fluorescence emitted by undoped and doped g-C_3_N_4_@HA microgel composites is observed. Boron (B), phosphorus (P), and sulfur (S) are the closest periodic table neighbors to carbon (C) and are frequently used as doping agents for various types of carbon-based materials, transforming them into useful paraphernalia for a wider range of applications [30,31]. The promotion of optical characteristics due to the nature of heteroatoms indicates that the main parameter for efficacy relates to the material’s electronic band structure [32]. Doping g-C_3_N_4_ with heteroatom elements is an effective procedure to improve optical characteristics, as this can affect electronic configurations in their carbonic framework structures and alter their surface reactivity [33]. Doping can change the levels of the highest occupied molecular orbital (HOMO) and lowest unoccupied molecular orbital (LUMO) in the structures. Monitoring fluctuations in band-gap energy levels caused by doping enables analysis of the role of these dopants in the structure in adjusting the optical properties of g-C_3_N_4_ [34]. It was reported that B-heteroatoms, as an n-type donor, can change the fluorescence properties of materials after doping with various interventions as they are more concentrated on the surface. P-heteroatoms, as an n-type donor, cause changes in fluorescence properties after doping by affecting the carbonic nucleus and electron–hole radiative recombination [35]. Also, the change in fluorescence properties caused by S doping, another n-type dopant, is due to the higher contribution of p-orbitals close to the Fermi level compared to s-orbitals [36]. These features are very promising for biomedical imaging, especially fluorescence diffuse optical tomography (FDOT)—a non-invasive technique based on localizing and quantifying exogenous fluorescent probes within tissues [37,38]. In particular, once injected into the body, the fluorescence emission from g-C_3_N_4_@HA microgels can be used to visualize the microgel distribution by FDOT, where the only limiting factor is the small tissue penetration depth of both UV excitation and visible fluorescent light that restricts their usage to lesions that are close to the skin surface [39,40]. It is also important to note that the employment of chemically stable g-C_3_N_4_ in FDOT to replace common fluorescent probes, i.e., dyes or fluorophore molecules, has significant advantages because it involves almost no photobleaching—the decay of fluorescence emission over time due to the photochemical alteration of these fluorescent molecules [41]. Nevertheless, the FDOT technique is limited by the extent of tissue heterogeneity and does not provide anatomical information; consequently, for functional image reconstruction, co-registration of techniques with spatial resolution such as computed tomography (CT) [42] or magnetic resonance imaging (MRI) is needed [43,44].

**Figure 3:**
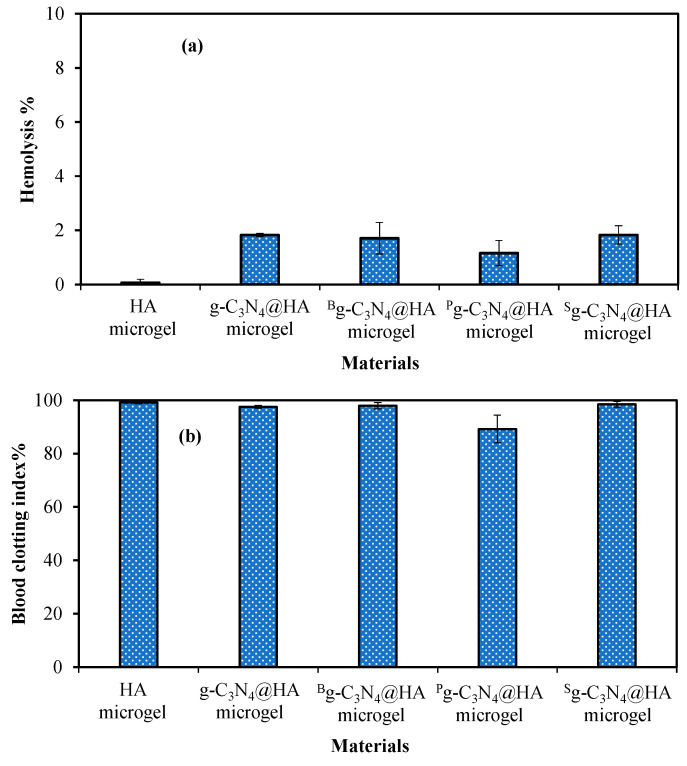
Blood compatibility of HA, g-C_3_N_4_@HA, ^B^g-C_3_N_4_@HA, ^P^g-C_3_N_4_@HA, and ^S^g-C_3_N_4_@HA microgels at 1 mg/mL concentration according to (**a**) hemolysis % and (**b**) blood clotting index % tests.

For the in vivo application of materials, blood compatibility of the materials needs to be considered seriously. Therefore, the hemolysis ratio and blood clotting index of the HA-based microgels were investigated to assess the toxicity of these materials in the blood. As given in Figure 3a, the hemolysis% (erythrocyte rupture %) was determined as 0.06 ± 0.03, 1.82 ± 0.06, 1.70 ± 0.58, 1.15 ± 0.46, and 1.82 ± 0.33% in the presence of 1000 µg/mL HA, g-C_3_N_4_@HA, ^B^g-C_3_N_4_@HA, ^P^g-C_3_N_4_@HA, and ^S^g-C_3_N_4_@HA microgels, respectively. It is clear that bare and g-C_3_N_4_@HA-based microgel composites were non-hemolytic materials with no significant destruction of red blood cells with hemolysis ratios below 2%. Materials with <5% hemolysis ratios are considered non-hemolytic.

The blood clotting values of HA, g-C_3_N_4_@HA, ^B^g-C_3_N_4_@HA, ^P^g-C_3_N_4_@HA, and ^S^g-C_3_N_4_@HA microgels at 1000 µg/mL concentration were 99.05 ± 0.36, 97.49 ± 0.53, 97.91 ± 1.19, 89.17 ± 5.20, and 98.40 ± 1.08%, as demonstrated in Figure 3b. Also, materials with a blood-clot index value >95% are considered viable, assuming no interference with clotting mechanisms in blood. These results show that none of the microgels affected the clotting mechanism of blood, suggesting that they can be securely used for in vivo blood contact applications. According to both hemocompatibility tests, HA and g-C_3_N_4_@HA microgel composites can be considered safe injectable materials for blood.

**Figure 4:**
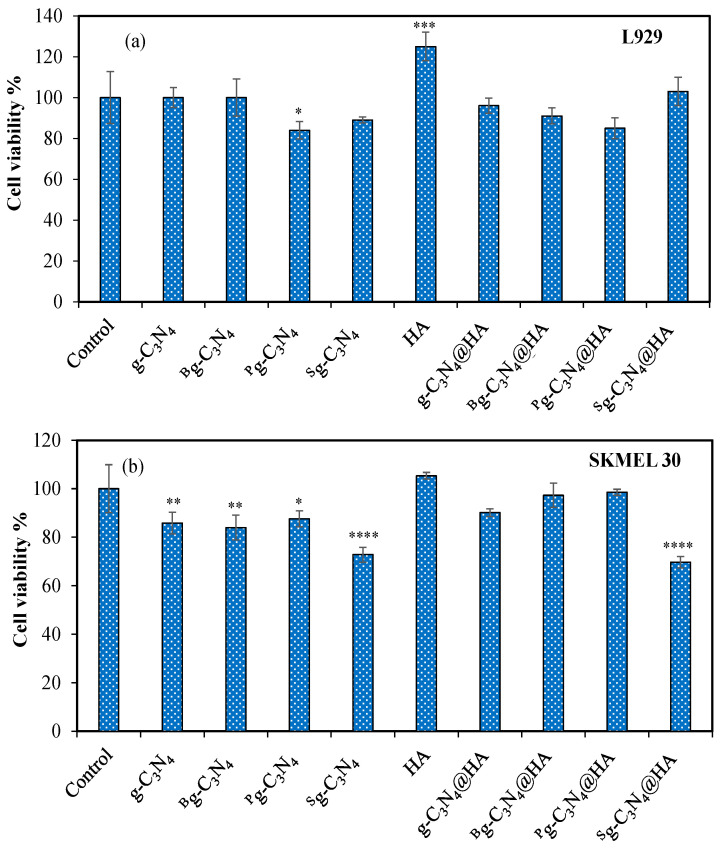
(**a**) Cytotoxicity of 100 μg/mL concentration of g-C_3_N_4_, ^B^g-C_3_N_4,_ ^P^g-C_3_N_4,_ and ^S^g-C_3_N_4_ and (**b**) 1000 μg/mL concentration of HA, g-C_3_N_4_@HA, ^B^g-C_3_N_4_@HA, ^P^g-C_3_N_4_@HA, and ^S^g-C_3_N_4_@HA microgels on L929 fibroblast cells and SKMEL 30 melanoma cells with 24 h incubation time. Statistical differences, p values are given as * < 0.05, ** < 0.01, *** < 0.001, and **** < 0.0001.

Furthermore, the cytotoxicity of these prepared g-C_3_N_4_-based structures and their HA microgel composites on L929 fibroblast cells and SKMEL 30 melanoma cells were also investigated during 24 h incubation time. The corresponding results are demonstrated in Figure 4. As shown in Figure 4a, g-C_3_N_4_ structures at 100 μg/mL concentration did not affect the cell viability of fibroblasts. The cell viability of the fibroblasts gradually increased by increasing the concentration of only HA microgels with a maximum 125 ± 7% cell viability value at 1000 µg/mL concentration. These results indicate that HA microgels improve the proliferation of healthy cells, pose no toxic effect, and can be used even in tissue engineering applications. In the literature, HA-based polymeric structures are generally reported as filler materials and biomimetic scaffolds for tissues in tissue engineering and cell therapy [45]. In addition, cell viability values of the fibroblasts in the presence of 1000 µg/mL concentrations of g-C_3_N_4_@HA, ^B^g-C_3_N_4_@HA, ^P^g-C_3_N_4_@HA, and ^S^g-C_3_N_4_@HA microgel composites were 96 ± 3%, 91 ± 4%, 85 ± 5%, and 103 ± 7%, respectively. As can be seen, there are no significant differences in the cytotoxicity of g-C_3_N_4_@HA-based microgel composites against fibroblasts, including for the composite forms of HA microgels with heteroatom-doped g-C_3_N_4_ structure, implying their potential for bioimaging applications using up to 1000 µg/mL concentration. 

The toxicity of these g-C_3_N_4_-based structures and their composite forms with HA microgels was also investigated on SKMEL 30 melanoma cells, and the results are given in Figure 4b. After 24 h incubation time, the cell viability % were 86 ± 5%, 84 ± 5%, 88 ± 3%, and 73 ± 3% at 100 μg/mL concentration of g-C_3_N_4_, ^B^g-C_3_N_4_, ^P^g-C_3_N_4_, and ^S^g-C_3_N_4_ structures, respectively. Furthermore, the cell viability of cancer cells in the presence of HA, g-C_3_N_4_@HA, ^B^g-C_3_N_4_@HA, ^P^g-C_3_N_4_@HA, and ^S^g-C_3_N_4_@HA microgels was determined as 105 ± 1%, 90 ± 2%, 97 ± 5%, 99 ± 1%, and 70 ± 2%, respectively. These results show that the heteroatom-doped g-C_3_N_4_ exhibited slight toxicity on cancer cells, but their composite forms with HA microgels exhibited somehow less toxicity against melanoma cells, except for ^S^g-C_3_N_4_@HA microgels. The statistical analysis supported these results with significant differences for g-C_3_N_4_-based materials and for only ^S^g-C_3_N_4_@HA composite microgels compared to the control group. 

**Figure 5:**
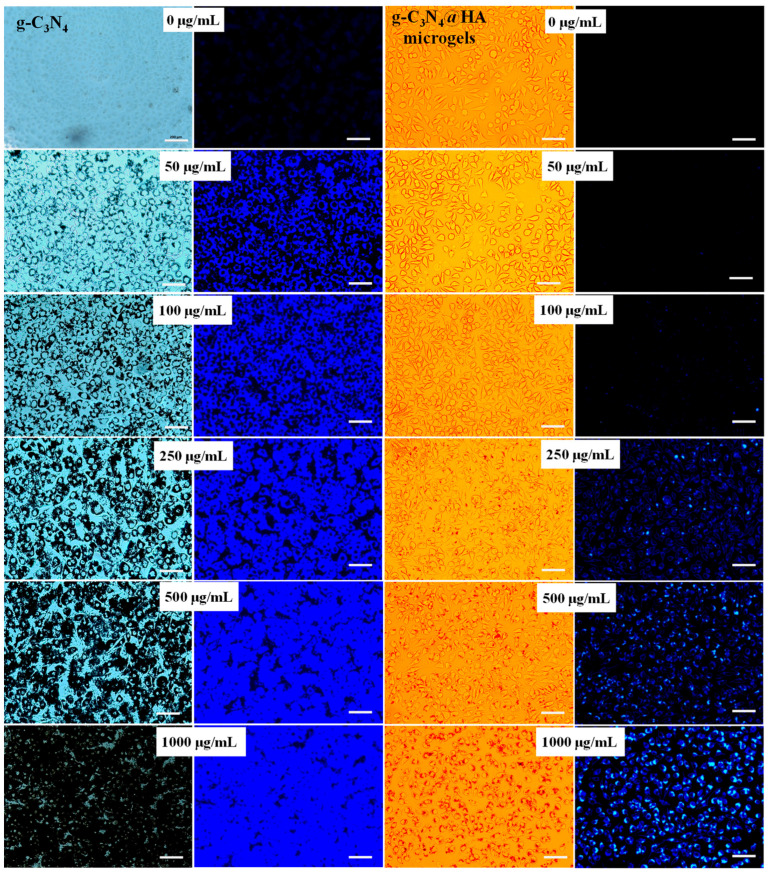
Light microscope and fluorescence microscope images of L929 fibroblast cells with different concentrations of g-C_3_N_4_ and g-C_3_N_4_@HA microgels under DAPI filter at 365 nm excitation during 24 h incubation time (After the incubation, the fibroblasts were washed with phosphate buffer solution (pH 7.4) three times to remove unattached materials). Scale bar is 200 μm.

The potential use of g-C_3_N_4_ and g-C_3_N_4_@HA microgels as fluorescence agents for cell imaging was examined using light and fluorescence microscope images of L929 fibroblasts after their interaction with various concentrations of these materials and 24 h incubation time. The results are given in Figure 5.

Before taking the image, the cells were washed with PBS (pH 7.4) three times to remove unattached g-C_3_N_4_@HA microgels. Light microscope images clearly show that only g-C_3_N_4_ structures could perfectly interact with cells at each concentration. However, at concentrations ≤100 µg/mL of g-C_3_N_4_, there are sufficient particles to provide perfect cell imaging without any material accumulation. Therefore, the morphology of the cells labeled with g-C_3_N_4_ up to 100 µg/mL concentration was largely visualized under the fluorescence microscope with a 365 nm DAPI filter. Bright blue images were obtained inside the cell and around the cell nuclei. In addition, the light microscope images show that g-C_3_N_4_@HA microgel composites slightly interacted with cells but did not provide enough intensity to observe cell images at 100 µg/mL concentration due to their removal with the washing process for the cells. However, the g-C_3_N_4_@HA microgel composite at a minimum 250 µg/mL concentration could provide good cell imaging. Similarly, with g-C_3_N_4_, g-C_3_N_4_@HA microgel composites at 1000 µg/mL concentration provided higher accumulated particles around the cell nuclei. Fluorescence images could be clearly visualized with 250–1000 µg/mL concentration ranges of g-C_3_N_4_@HA microgel composite, and the highest intensity image was obtained at 1000 µg/mL concentration of microgel composites. In the synthesis process, 1000 µg g-C_3_N_4_@HA microgel composite was prepared using 100 µg concentration of g-C_3_N_4_. The amount of g-C_3_N_4_ inside the microgel composite was determined by measuring the fluorescence intensity of the materials against the calibration curve for the g-C_3_N_4_ suspension. The results are summarized in Table 1. According to the fluorescence intensity analysis, 1000 µg of g-C_3_N_4_@HA microgel composite contained only 63.6 µg of g-C_3_N_4_.

Therefore, fluorescence intensity significantly decreased for g-C_3_N_4_@HA microgel composite compared with only g-C_3_N_4_ at the same concentration. In addition, fibroblast cells did not exhibit nuclear staining by g-C_3_N_4_, which is similar to g-C_3_N_4_@HA microgel composite, but a bright blue fluorescence image was observed around the cell nuclei and inside the cells using both materials. 

**Figure 6:**
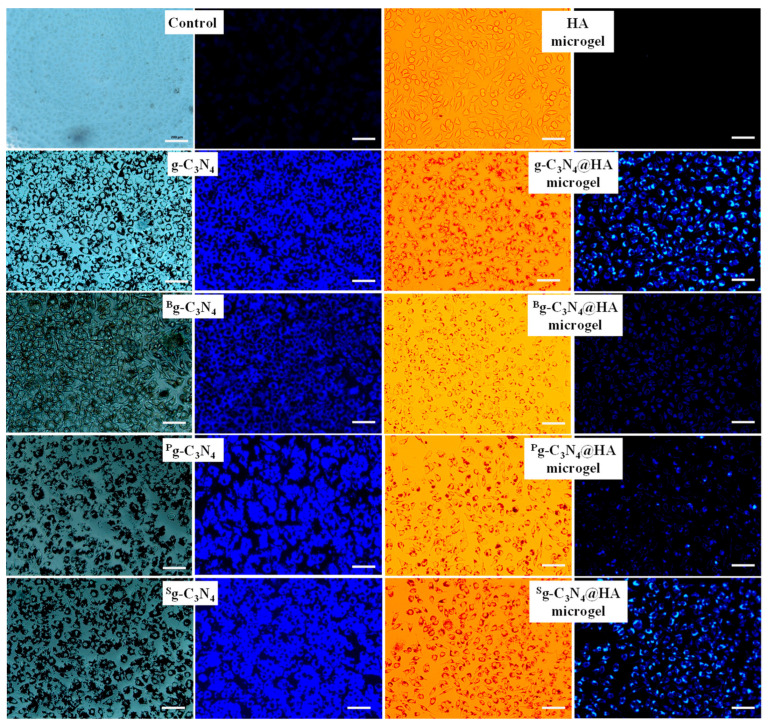
Light microscope and fluorescence microscope images of L929 fibroblast cells treated with 100 μg/mL concentration of g-C_3_N_4_, ^B^g-C_3_N_4_, ^P^g-C_3_N_4_, and ^S^g-C_3_N_4_ and 1000 μg/mL concentration of HA, g-C_3_N_4_@HA, ^B^g-C_3_N_4_@HA, ^P^g-C_3_N_4_@HA, and ^S^g-C_3_N_4_@HA microgels under DAPI filter at 365 nm excitation after 24 h incubation time (After the incubation process, the fibroblasts were washed with phosphate buffer solution (pH 7.4) three times to remove unattached materials). Scale bar is 200 μm.

To investigate the effects of heteroatom doping of g-C_3_N_4_ and their HA microgel composite forms on cell imaging applications, the microscope images of fibroblasts were taken with 100 μg/mL g-C_3_N_4_-based materials and 1000 μg/mL microgel composites. The results are presented in Figure 6. All forms of g-C_3_N_4_ provide excellent fluorescence imaging with bright blue cell images under the DAPI filter at 365 nm. Furthermore, g-C_3_N_4_ and ^B^g-C_3_N_4_ provided good cell imaging when compared with the other heteroatom-doped forms because of good distribution without any accumulation on/inside the cells. All types of g-C_3_N_4_ @HA microgels can readily interact and penetrate inside the cells, as seen with light microscope imaging. However, only 1000 μg/mL g-C_3_N_4_ @HA and ^S^g-C_3_N_4_ @HA microgel composites could be used for fluorescence imaging of cells due to high fluorescence intensity in comparison to the images obtained using ^B^g-C_3_N_4_@HA and ^P^g-C_3_N_4_@HA microgel composites. Fluorescence intensity differences between heteroatom-doped g-C_3_N_4_ with their microgel forms indicate that 63.6, 17.1, 23.1, and 14.9 µg of g-C_3_N_4_, ^B^g-C_3_N_4_, ^P^g-C_3_N_4_, and ^S^g-C_3_N_4_ were embedded into 1000 µg HA microgel network, respectively, as listed in Table 1. These results support the fluorescence microscope images of the materials, which indicate lower fluorescence intensity compared to the g-C_3_N_4_ forms. For in vivo bioimaging applications, autofluorescence, which can arise from endogenous fluorophores, may overlap, and generate problems for the fluorescence properties of the materials that are below the near-infrared range. As g-C_3_N_4_ nanosheets are quite bright and have high fluorescence intensity, the autofluorescence effect might not be significant under a blue filter. On the other hand, ^B^g-C_3_N_4_@HA and ^P^g-C_3_N_4_@HA microgel composites have low fluorescence intensity, which may be cumbersome for in vivo imaging applications. As reported by Urandur et al., the autofluorescence wavelength of the cells could be overcome by modification or conjugation of the materials with NIR fluorescent dyes to shift the fluorescence intensity in the NIR range, allowing in vivo bioimaging over a wide range of wavelengths [46]. 

**Figure 7:**
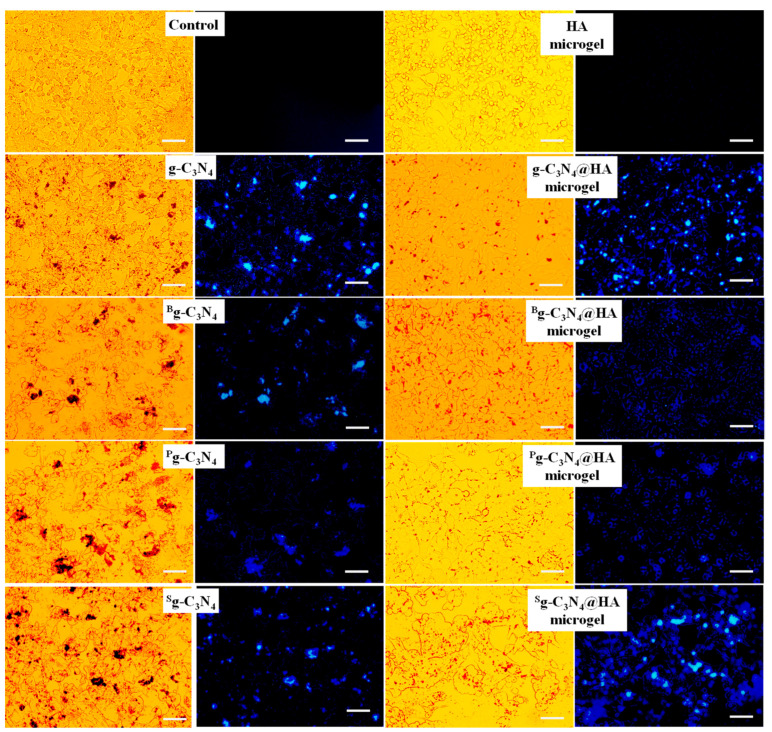
Light microscope and fluorescence microscope images of SKMEL 30 skin-cancer cells treated with 100 μg/mL concentration of g-C_3_N_4_, ^B^g-C_3_N_4_, ^P^g-C_3_N_4_, and ^S^g-C_3_N_4_ and 1000 μg/mL concentration of HA, g-C_3_N_4_@HA, ^B^g-C_3_N_4_@HA, ^P^g-C_3_N_4_@HA, and ^S^g-C_3_N_4_@HA microgels under DAPI filter at 365 nm excitation at 24 h incubation time (After the incubation process, the cells were washed with phosphate buffer solution (pH 7.4) three times to remove unattached materials). Scale bar is 200 μm.

The light and fluorescence microscope images for SKMEL 30 melanoma cells in the presence of 100 μg/mL concentration of g-C_3_N_4_-based materials and 1000 μg/mL concentration of HA, g-C_3_N_4_, ^B^g-C_3_N_4_, ^P^g-C_3_N_4_, and ^S^g-C_3_N_4_ microgels were visualized and given in Figure 7.

g-C_3_N_4_ and its heteroatom-doped forms were not attached to the melanoma cells, and many were removed during the washing process with PBS. Light microscope images observed that the g-C_3_N_4_-based materials remaining after washing accumulated on some parts of the cells and were not well dispersed on the cancerous cells. In addition, the fluorescence intensity of the g-C_3_N_4_-based materials and their microgel composites in the fluorescence images were similar because of the low interaction of the g-C_3_N_4_ forms with the tumorous cells. It can be said that g-C_3_N_4_-based materials did not interact with SKMEL 30 melanoma cells effectively for imaging purposes. The g-C_3_N_4_@HA microgel composites interacted with SKMEL 30 melanoma cells very well, with good distribution according to the light and fluorescence microscope images of the cells treated with g-C_3_N_4_@HA-based microgel composites. It is worth noting that some HA can bind to receptors such as CD44 for hyaluronic acid-mediated motility (RHAMM), P-selectin, and lymphatic vessel endocytic receptor (LYVE-1), which are common on malignant cells in comparison with healthy cells [16,17,18,19]. Therefore, HA-based polymeric materials are generally used as cancer drug carriers in targeted cancer therapy [18]. In this study, the targeting potential of the g-C_3_N_4_@HA microgel composites for SKMEL 30 melanoma cells was expressed by fluorescence imaging variations of only g-C_3_N_4_ and their HA microgel composites. These results indicate that g-C_3_N_4_@HA-based microgel composites have great potential as targeting carrier materials for cancer therapy, in addition to their bioimaging visualization capability. 

**Figure 8:**
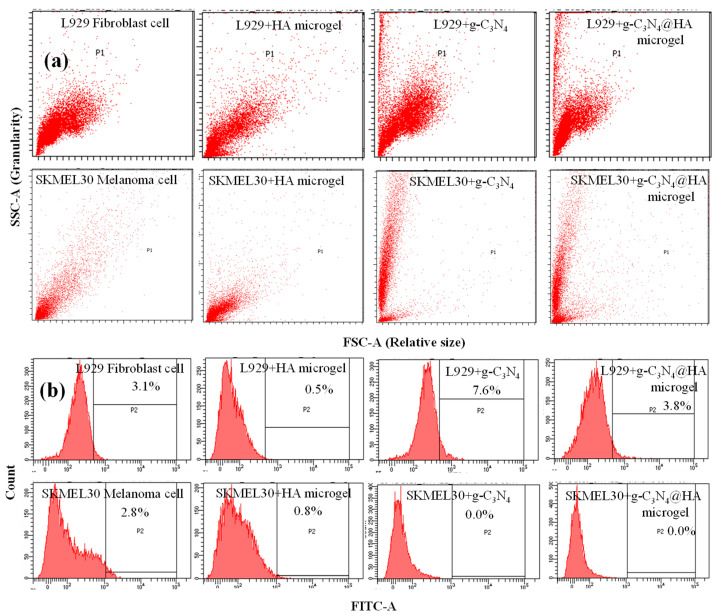
(**a**) Granularity-relative size graphs and (**b**) green fluorescence from flow cytometry analyses of L929 fibroblast cells and SKMEL 30 melanoma cells interacting with HA microgel, g-C_3_N_4_, and g-C_3_N_4_@HA microgels after 18 h incubation.

The viability of L929 fibroblast cells and SKMEL 30 melanoma cells and their distribution in the presence of HA microgel, g-C_3_N_4_, and g-C_3_N_4_@HA microgel composites was monitored by flow cytometry. The results of the analyses are given in Figure 8a,b.

According to the granularity versus relative size graphs of the cells, the distributions of the fibroblast and melanoma cells were linear. Similarly, HA microgel interacting with the cells had almost the same distribution, as there was no significant toxicity induced by HA microgels. Granularity graphs of fibroblast cells in the presence of g-C_3_N_4_ and g-C_3_N_4_@HA microgels show two populations because of the smaller size and granularity of these materials compared to the cells. Furthermore, g-C_3_N_4_ and g-C_3_N_4_@HA microgels exhibited the distinct material size distribution of the melanoma cell distribution to a lesser degree. Fluorescence and viable cell count for fibroblast and melanoma cells in the presence of HA microgel, g-C_3_N_4_, and g-C_3_N_4_@HA microgels were obtained under FITC-A (green) without any fluorescence staining, and results are presented in Figure 8b. It is obvious from Figure 8b that HA microgels did not stain any cells with 0.5% cell count as expected. Notably, the fluorescence and viable cells for fibroblasts treated with g-C_3_N_4_ and g-C_3_N_4_@HA microgels resulted in much higher fluorescence cell counts than bare HA microgel with values of 7.6% and 3.8%, respectively. These values are too low in comparison with fluorescence microscope images of these materials. This is because these results were not obtained from blue fluorescence, which is the fluorescence property of g-C_3_N_4_-based materials. Similarly, the melanoma cell count was 0.8% for HA microgels, but interestingly, no viable cancer cells were detected in the presence of g-C_3_N_4_ and g-C_3_N_4_@HA microgels. These results show that HA microgel is a non-toxic material when interacting with fibroblast and melanoma cells. In addition, g-C_3_N_4_ and g-C_3_N_4_@HA microgels were non-toxic for fibroblast cells, but these materials significantly affected the viability of cancer cells. As reported, reactive oxygen species (ROS), which can be generated by the photoactivated fluorescence of g-C_3_N_4_, could inhibit the growth and progress of cancerous cells for potential photocatalytic cancer therapy [47]. Therefore, it can be inferred that cancer-cell inhibition by g-C_3_N_4_ and g-C_3_N_4_@HA microgel composites depends on the ROS generation potency of g-C_3_N_4_. Consequently, g-C_3_N_4_@HA-based microgel composites can be used in cancer treatment due to the light activation ability of g-C_3_N_4_ without any toxicity on healthy cells. 

## 3. Materials and Methods

### 3.1. Materials

Sodium hyaluronate (HA, MW:1.5–2.2 MDa, 95% Acros Organics), divinyl sulfone (DVS, 97%, Merck, Darmstadt, Germany), sodium bis(2-ethylhexyl) sulfosuccinate (AOT, 96%, Acros Organics, Geel, Belgium), isooctane (≥99.5%, Isolab, Eschau, Germany), and acetone (99%, BRK, Istanbul, Turkey) were used in the synthesis of HA microgels. Melamine (99%, Sigma–Aldrich, St. Louis, MO, USA) was used as a precursor to synthesize the graphitic carbon nitride (g-C_3_N_4_) structures. Boric acid (99.5%, Sigma–Aldrich, St. Louis, MO, USA), phosphorus red (97%, Merck, Italy), and sulfur (Reagent grade, Sigma–Aldrich, St. Louis, MO, USA) were used as B, P, and S sources for doping of the g-C_3_N_4_ structures. The L929 fibroblast cells (Mouse C3, An2 connective tissue, HUKUK No: 92123004) and SKMEL 30 An1 human melanoma cells (HUKUK No: 03010901) were obtained from the Culture Collection of Animal Cells, HUKUK, SAP institute, Ankara, Turkey. For cytotoxicity and bioimaging analysis, Dulbecco’s Modified Eagle’s Medium (DMEM, with 4.5 g/L glucose, 3.7 g/L sodium pyruvate, L-glutamine 0.5 g/mL), RPMI-1640 (2 mM L-glutamine, 1 mM sodium pyruvate, 4.5 g/L glucose, 10 mM HEPES, 1.5 g/L NaCO3), fetal bovine serum (FBS, heat inactivated), and penicillin/streptomycin (10,000 U/mL penicillin, 10 mg/mL streptomycin) were purchased from Panbiotech (Aidenbach, Germany). Dimethyl sulfoxide (DMSO, Carlo Erba, 99.9%, France) and 3-(4,5-dimethylthiazol-2-yl)-2,5-diphenyltetrazolium bromide (MTT agent, 98%, neofroxx, Einhausen, Germany) were used in the cytotoxicity test. High-purity DI water was obtained from Millipore-Direct Q UV3 (Merck Darmstadt, Germany) at 18.2 M.Ω.cm. 

### 3.2. Preparation of Bare HA Microgels and g-C_3_N_4_@HA-Based Microgel Composites

The synthesis of g-C_3_N_4_ and their heteroatom-doped forms was reported by our group in a previous study [20]. For microgel preparation, the HA solution was prepared at 15 mg/mL concentration in 10 mL of 0.1 M NaOH aqueous solution. Separately, the g-C_3_N_4_ suspension was prepared at 15 mg/mL concentration in 1 mL DI water with a 3 h sonication process. Then, the g-C_3_N_4_ suspensions were placed into the HA solution by stirring at 500 rpm for 15 min, and 1.1 mL of this suspension was dispersed in 0.2 M AOT/isooctane emulsion medium under a stirring rate of 1000 rpm. After 15 min, DVS as crosslinker at 25 mol.% relative to HA repeating unit was placed in the microemulsion medium and stirred for 1 h at 1000 rpm. At the end of the reaction, the prepared g-C_3_N_4_@HA-based microgel composites were precipitated in an excess amount of acetone. The precipitated g-C_3_N_4_@HA-based microgel was washed with acetone:water (90:10, *v*:*v*) solution two times and only acetone two times using a centrifuge at 10,000 rpm for 5 min. After that, the clean g-C_3_N_4_@HA-based microgel was dried in an oven at 50 °C and kept in a closed container for further applications. Only HA microgel was synthesized by the same process without using g-C_3_N_4_ suspension.

### 3.3. Characterization of g-C_3_N_4_-Based Materials and g-C_3_N_4_@HA-Based Microgel Composites

The scanning electron microscopy (SEM) images of g-C_3_N_4_ were obtained by SEM (Hitachi Ultra High-Resolution Analytical, FE-SEM SU-70, Tokyo, Japan) at a 15 kV operating voltage. The samples of sonicated and dried g-C_3_N_4_ were placed on SEM stubs using carbon tape and coated with Au for 10 s before acquiring images.

The morphological structure of HA microgels and composite forms with g-C_3_N_4_ were visualized by scanning electron microscope (SEM, QUANTA 400F Field Emission) at 20 kV operating voltage. The samples for SEM imaging were prepared by suspending the microgels in ethanol, placing a drop of the microgel suspensions on the SEM stab, and evaporating the solvent (ethanol) before coating with Au/Pb for 10 s. 

X-ray Photoelectron Spectroscopy (XPS, Thermo Fisher Scientific K-Alpha) measurements were carried out with an XPS system equipped with a micro-focus X-ray source using aluminum (Al) K-α characteristic radiation (spot size 400 μm) and 180o double-focusing hemispherical analyzer detector. The survey spectrum was obtained in the range 0–1350 eV with an energy step of 1 eV and 15 scans, while the individual peaks were scanned with an energy step of 0.1 eV, collecting 10 scans. The peak labeling and theoretical fits of the peaks were done using Advantage Software (Stuart, FL, USA), which is built into the XPS system. 

The Fourier transform infrared (FT-IR, Nicolet iS10, Thermo, MA, USA) spectrum of prepared bare and g-C_3_N_4_-based structures embedded in HA microgels was recorded between 4000 and 650 cm^−1^ using an attenuated total reflector device.

The thermal behaviors of prepared HA and g-C_3_N_4_@HA-based microgels were investigated using a thermogravimetric analyzer (TGA, DT/TG 6300, Exstar, Seiko, Japan). For this purpose, first, the moisture in the microgels was removed by heating up to 100 °C under N_2_ gas at a flow rate of 200 mL/min. After that, the thermal behaviors of microgels were investigated with heating up to 750 °C from 100 °C at 10 °C/min heating rate under N_2_ gas at a flow rate of 200 mL/min.

Steady-state fluorescence spectra of doped and undoped g-C_3_N_4_@HA microgels were obtained using a Fluorescence Spectrometer FLSP920 (Edinburgh Instruments, Livingston, UK) equipped with 450 W Xe900 xenon lamp as an excitation source and single photon counter/photomultiplier tube as the detector. Measurements were performed at room temperature on microgels diluted with pure water to increase the transparency. Excitation spectra were recorded by scanning the wavelength range λ = 200–432 nm, while emission spectra were recorded between 406 and 688 nm in 2 nm steps with a 5 nm scan slit.

The amounts of g-C_3_N_4_-based structures embedded in HA microgels were also determined with a fluorescence spectrophotometer using prepared calibration curves. For this purpose, the calibration curves for bare g-C_3_N_4_ and heteroatom-doped ^B^g-C_3_N_4_, ^P^g-C_3_N_4_, and ^S^g-C_3_N_4_ structures were prepared at concentration ranges of 6–100 µg/mL. After that, the amounts of g-C_3_N_4_-based structures embedded within HA microgels were determined from the fluorescent intensity obtained from g-C_3_N_4_@HA-based microgel suspensions at 1 mg/mL concentrations. 

### 3.4. Blood Compatibility of g-C_3_N_4_@HA-Based Microgel Composites

Blood toxicity of HA, g-C_3_N_4_@HA, ^B^g-C_3_N_4_@HA, ^P^g-C_3_N_4_@HA, and ^S^g-C_3_N_4_@HA microgels at 1000 µg/mL concentration was determined using hemolysis and blood compatibility tests with human blood. The hemolysis and blood clotting assays were applied based on the method described by Sahiner et al. (2022) [28]. For the analysis, human blood was taken from healthy volunteers after permission from Canakkale Onsekiz Mart University, Human Research Ethics Committee (2011-KAEK-27/2022). 

### 3.5. Cytotoxicity Analysis of g-C_3_N_4_-Based Materials and g-C_3_N_4_@HA-Based Microgel Composites

Cell toxicity effects of g-C_3_N_4_-based materials and g-C_3_N_4_@HA-based microgel composites on healthy L929 fibroblast cells and SKMEL 30 melanoma cells were determined using in vitro cytotoxicity analysis with MTT assay according to the procedure proposed by Sahiner et al. (2022) [28]. Confluent fibroblast cells at 5 × 10^4^ cells/well in DMEM + FBS medium and melanoma cells at 5 × 10^3^ cells/well in RPMI + FBS medium were cultured in 96-well plates and incubated at 37 °C with 5% CO_2_ atmosphere for 24 h. Then, the attached cells interacted with 100 µg/mL concentration of g-C_3_N_4_-based materials and 1000 µg/mL concentration of g-C_3_N_4_@HA=based microgel composites prepared in the growth medium at the same conditions for 24 h more. After that, the material suspension was removed, and the cells were washed with phosphate buffer solution (PBS) three times. Then, the cells were incubated with 100 µL 0.5 mg/mL concentration of MTT reagent for 2 h in the dark. At the end of the incubation, the MTT agent was removed from the wells, and 200 µL of DMSO was placed into the wells to dissolve formazan crystals. Finally, the absorbance was recorded at 570 nm with a plate reader (Thermo, Multiskan Sky, Themo, MA, USA). All experiments were performed three times, and results were given with standard deviations. For statistical analysis, one-way ANOVA and Dunnett’s multiple-comparison test were performed using GraphPad Prism (Version 9 software), and a *p*-value below 0.05 was considered a significant difference compared with the control group. 

### 3.6. Bioimaging Properties of g-C_3_N_4_-Based Materials and g-C_3_N_4_@HA-Based Microgel Composites

To investigate the potential use in cell imaging applications, 100 µg/mL concentration of g-C_3_N_4_-based materials and 1000 µg/mL concentration of g-C_3_N_4_@HA-based microgel composites were interacted with healthy L929 fibroblast cells and SKMEL 30 melanoma cells for 24 h incubation in a 96-well plate after the culture process described in the cytotoxicity analysis above. Then, the cells were washed with PBS three times to remove unattached materials and visualized by light microscope and fluorescence microscope (ZEISS, Axioscope, Oberkochen, Germany) under a DAPI filter at 365 nm. 

### 3.7. Flow Cytometry Analysis of HA Microgel, g-C_3_N_4_, and g-C_3_N_4_@HA-Based Microgel Composites

Cell distribution and viability of L929 fibroblast cells and SKMEL 30 melanoma cells in the presence of HA microgel, g-C_3_N_4_, and g-C_3_N_4_@HA-based microgel composites was investigated by means of flow cytometry analysis. Cell suspensions of 5 × 10^4^ cells in a sterile 0.9% NaCl aqueous solution were used. Separately, 1 mg/mL concentration of HA microgel, g-C_3_N_4_, and g-C_3_N_4_@HA microgel composites suspended in 0.9% NaCl aqueous solution were mixed with these cell suspensions gently at a 1:1 volume ratio. After 18 h incubation, the suspension was analyzed by flow cytometry (BD LSRFortessa Cell Analyzer) without any staining, and about 10,000 events were collected for all cells.

## 4. Conclusions

Here, the synthesis and characterization of heteroatom-doped g-C_3_N_4_ embedded HA microgels were accomplished. The band-gap energies of the composites were estimated as Eg = 2.75 eV for undoped g-C_3_N_4_ and the same (2.75 eV) for ^S^g-C_3_N_4_, as well as Eg = 2.66 eV for P-doped and Eg = 3.0 eV for B-doped g-C_3_N_4_. HA microgel composites were prepared as ^H^g-C_3_N_4_@HA (H: S, P, or B) for cancer-cell imaging and destruction via photoactivation. The g-C_3_N_4_@HA-based microgel composites are safe materials because of excellent blood compatibility assessed by hemolysis% and blood clotting index% and almost no cytotoxicity on healthy fibroblast cells confirmed by MTT assay. These studies show that heteroatom-doped g-C_3_N_4_ has significant penetration ability into healthy fibroblast cells as a fluorescence imaging agent but does not have good distribution and interaction with skin-cancer cells. The low interaction ability of heteroatom-doped g-C_3_N_4_ with melanoma cells was overcome by the preparation of the composite heteroatom-doped g-C_3_N_4_ with HA microgels to take advantage of the well-known targeting ability of HA for cancer cells via different receptors that are expressed on the cancerous cells. This was corroborated by the fluorescence imaging of SKMEL 30 melanoma cells. It was clearly shown that the fluorescence properties of heteroatom-doped g-C_3_N_4_, in combination with the cancer-cell targeting ability of HA for the prepared ^H^g-C_3_N_4_@HA, along with their photoactivation capacity, can provide not just diagnostic but also theragnostic features with superior therapeutic effect.

## Figures and Tables

**Table 1:** Amount of ^H^g-C_3_N_4_ embedded within HA microgel composites.

Microgel Composites	g-C_3_N_4_ Amounts in HA Microgels (µg/mg)
g-C_3_N_4_@HA	127.2
^B^g-C_3_N_4_@HA	34.1
^P^g-C_3_N_4_@HA	46.2
^S^g-C_3_N_4_@HA	29.9

## Data Availability

Data are contained within the article.

## Supplementary Materials

The following supporting information can be downloaded at: https://www.mdpi.com/article/10.3390/ph17020160/s1, Figure S1: The schematic presentation of synthesis of (a) g-C_3_N_4_ and heteroatom-doped g-C_3_N_4_ (^H^g-C_3_N_4_, H: B, P, S), (b) g-C_3_N_4_ embedded HA microgels (g-C_3_N_4_@HA microgel), and (c) digital camera images of prepared HA and g-C_3_N_4_@HA microgels; Figure S2: (a) The XPS spectra of undoped g-C_3_N_4_@HA microgels obtained by survey scan and (b) theoretical fits for high-resolution C 1S, and (c) N 1S peaks showing the decomposition into individual contributions from different atoms; Figure S3: (a) FT-IR spectrum, and (b) TGA thermograms of prepared g-C_3_N_4_ structures embedded HA microgels.
