# Supplementary material for: Fluorescent Graphitic Carbon Nitride (g-C3N4)-Embedded Hyaluronic Acid Microgel Composites for Bioimaging and Cancer-Cell Targetability as Viable Theragnostic
Source: Pharmaceuticals (Basel). 2024 Jan 25;17(2):160. doi: 10.3390/ph17020160 (PMC10893513; doi:10.3390/ph17020160)
Supplement: Supplementary file 1 [file pharmaceuticals-17-00160-s001.zip › pharmaceuticals-2780394-supplementary.pdf]

Article

Fluorescent Graphitic Carbon Nitride ($g-C_3N_4$)-Embedded Hyaluronic Acid Microgel Composites for Bioimaging and Cancer-Cell Targetability as Viable Theragnostic

Selin S. Suner [1], Mehtap Sahiner [2], Sahin Demirci [1], Evrim Umut [3,4] and Nurettin Sahiner [1,5,6,*]

[1] Department of Chemistry, Faulty of Science, Canakkale Onsekiz Mart University, 17100 Canakkale, Turkey; sagbasselin@gmail.com (S.S.S.); sahindemirci@gmail.com (S.D.)
[2] Department of Bioengineering, Faculty of Engineering, Canakkale Onsekiz Mart University Terzioglu Campus, 17100 Canakkale, Turkey; sahinerm78@gmail.com
[3] Department of Medical Imaging Techniques, School of Healthcare, Dokuz Eylul University, 35330 Izmir, Turkey; evrim.umut@deu.edu.tr
[4] BioIzmir-Izmir Health Technologies Development and Accelerator Research and Application Center, Dokuz Eylul University, 35330 Izmir, Turkey
[5] Department of Chemical and Biomolecular Engineering, University of South Florida, Tampa, FL 33620, USA
[6] Department of Ophthalmology, Morsani College of Medicine, University of South Florida, Tampa, FL 33612, USA
* Correspondence: sahiner71@gmail.com or nsahiner@usf.edu

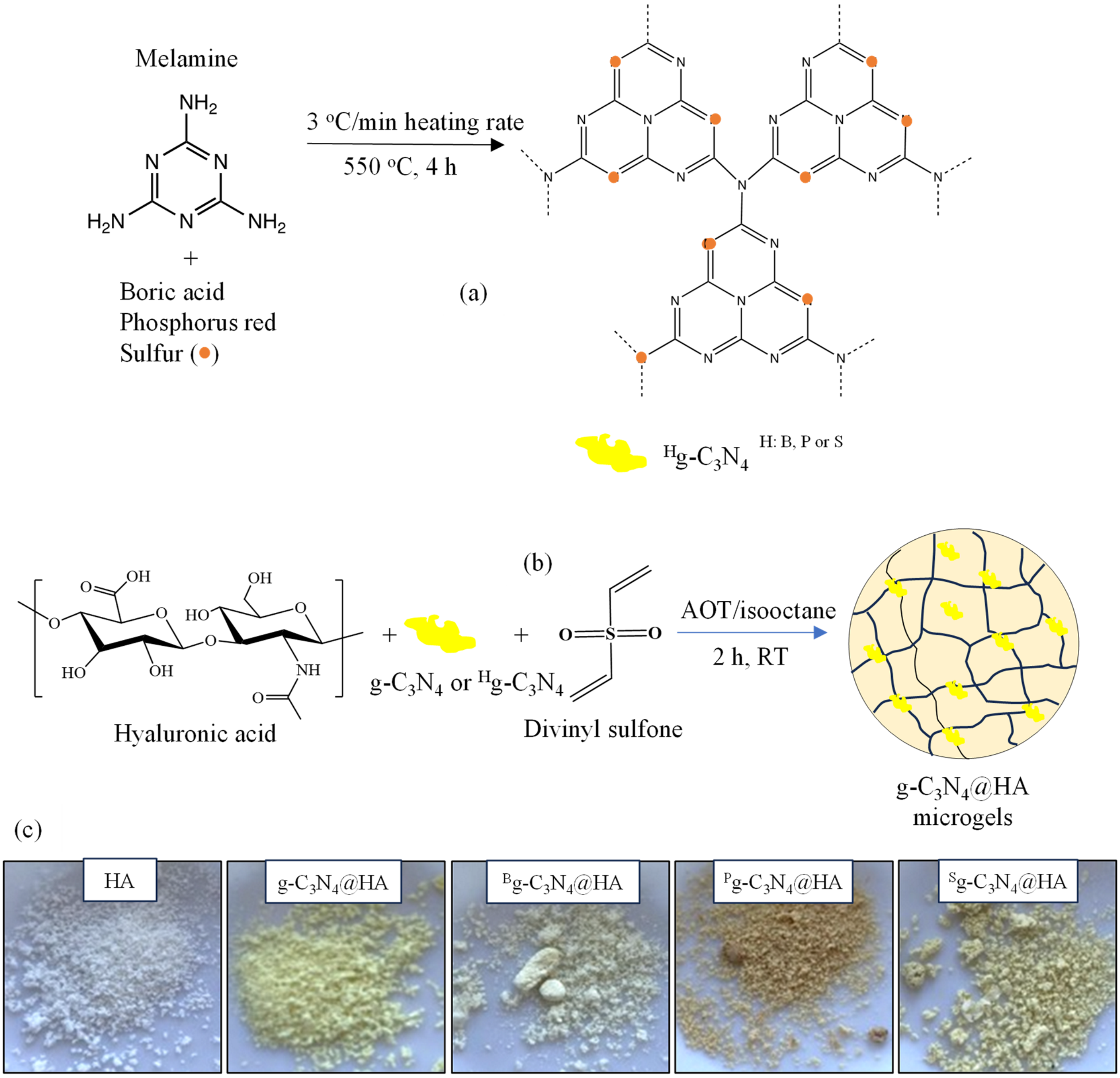

Figure S1. The schematic presentation of synthesis of **(a)** g-C_3N_4, and heteroatom doped g-C_3N_4 (^{H}g-C_3N_4, H: B, P, S), **(b)** g-C_3N_4 embedded HA microgels (g-C_3N_4@HA microgel), and **(c)** digital camera images of prepared HA and g-C_3N_4@HA microgels.

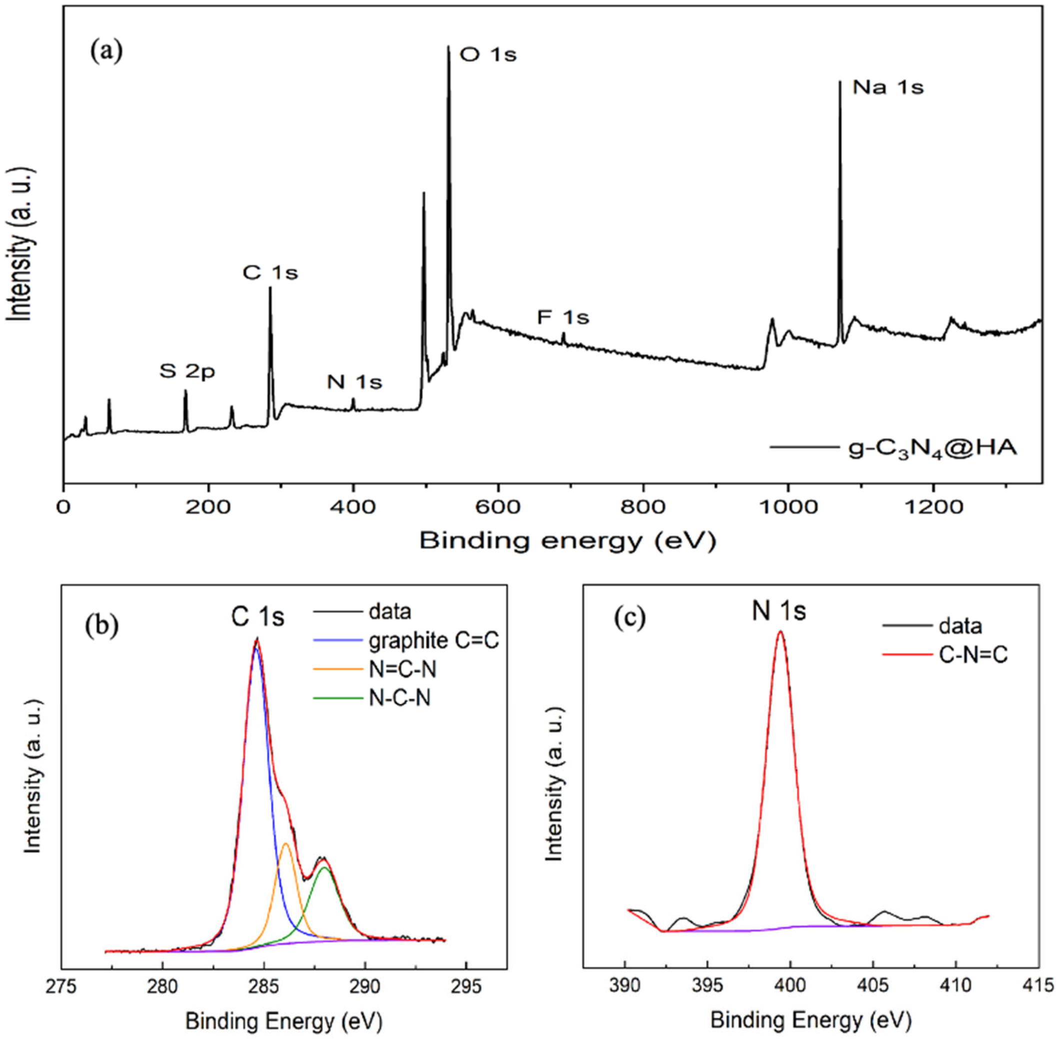

Figure S2. (a) The XPS spectra of undoped g-C_3N_4@HA microgels obtained by survey scan and **(b)** theoretical fits for high resolution C 1S, and **(c)** N 1S peaks showing the decomposition into individual contributions from different atoms.

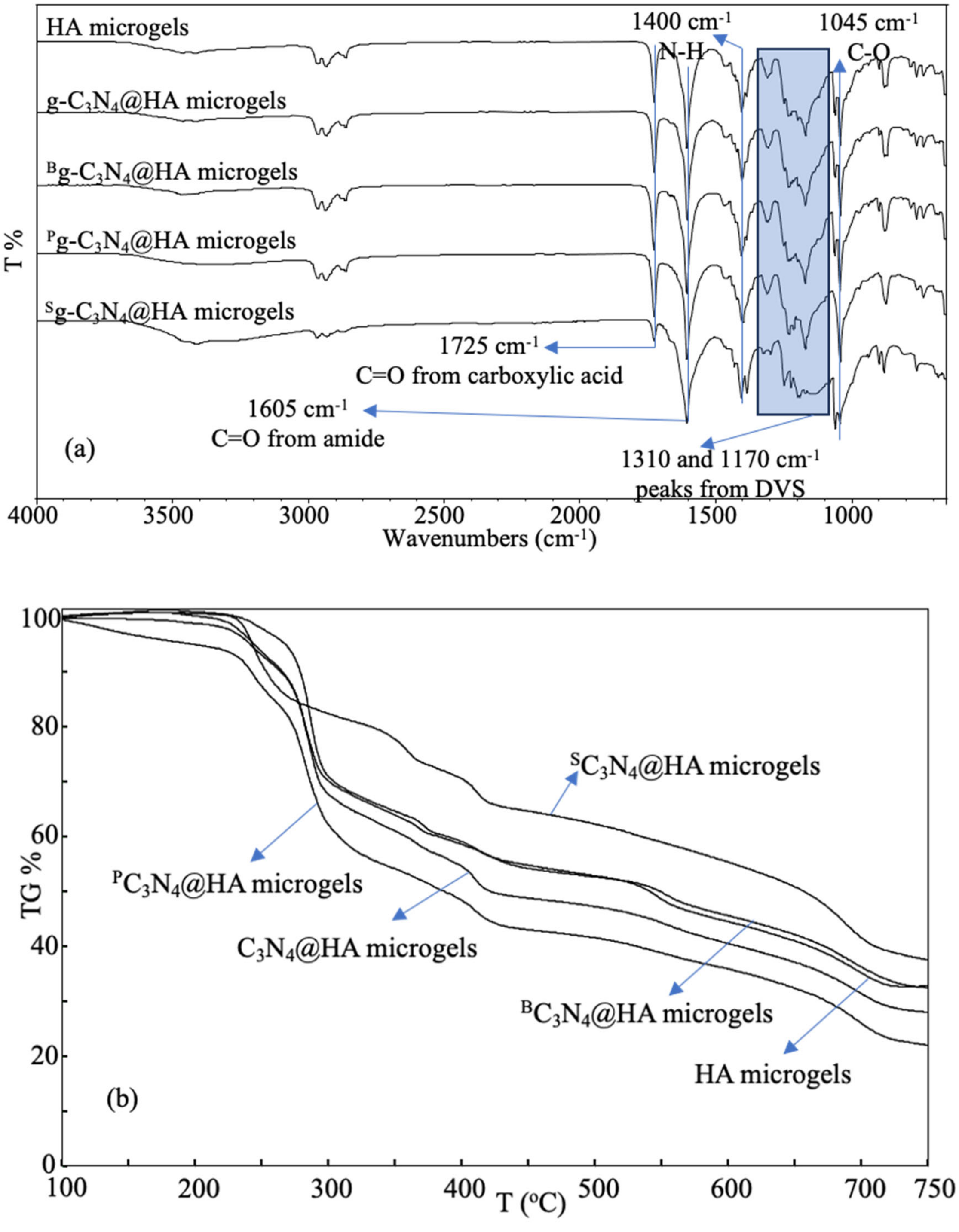

Figure S3. (a) FT-IR spectrum, and **(b)** TGA thermograms of prepared g-C_3N_4 structures embedded HA microgels.
